# Knowledge translation tools to guide care of non-intubated patients with acute respiratory illness during the COVID-19 Pandemic

**DOI:** 10.1186/s13054-020-03415-2

**Published:** 2021-01-08

**Authors:** David Leasa, Paul Cameron, Kimia Honarmand, Tina Mele, Karen J. Bosma, for the LHSC Ventilation Strategy for COVID-19 Working Group, Rob Arntfield, John Basmaji, Karen J. Bosma (lead), Paul Cameron, Ian Dashnay, Rob Ducharme, Belinda Gougoulias, Jeff Granton, Wael Haddara, Ahmed F. Hegazy, Kimia Honarmand, Kendrah Krouskos, John Landau, David Leasa, Rob Leeper, Christine MacDonald, Claudio Martin, David McCormack, Rachelle McCready, Tina Mele, Brenda Morgan, Dave Nagpal, Ruediger Noppens, Allison Oldershaw, Marat Slessarev, Joanne Smith, Michelle Stephens, Ravi Taneja, Susan Whitehouse, Tim Winterburn

**Affiliations:** 1 London Health Sciences Centre, 339 Windermere Road, London, ON N6A 5A5 Canada; 2 Western University, 1151 Richmond St, London, ON N6A 3K7 Canada

**Keywords:** COVID-19, Non-invasive ventilation, High flow nasal oxygen, Prone positioning, ROX index

## Abstract

Providing optimal care to patients with acute respiratory illness while preventing hospital transmission of COVID-19 is of paramount importance during the pandemic; the challenge lies in achieving both goals simultaneously. Controversy exists regarding the role of early intubation versus use of non-invasive respiratory support measures to avoid intubation. This review summarizes available evidence and provides a clinical decision algorithm with risk mitigation techniques to guide clinicians in care of the hypoxemic, non-intubated, patient during the COVID-19 pandemic. Although aerosolization of droplets may occur with aerosol-generating medical procedures (AGMP), including high flow nasal oxygen and non-invasive ventilation, the risk of using these AGMP is outweighed by the benefit in carefully selected patients, particularly if care is taken to mitigate risk of viral transmission. Non-invasive support measures should not be denied for conditions where previously proven effective and may be used even while there is suspicion of COVID-19 infection. Patients with de novo acute respiratory illness with suspected/confirmed COVID-19 may also benefit. These techniques may improve oxygenation sufficiently to allow some patients to avoid intubation; however, patients must be carefully monitored for signs of increased work of breathing. Patients showing signs of clinical deterioration or high work of breathing not alleviated by non-invasive support should proceed promptly to intubation and invasive lung protective ventilation strategy. With adherence to these principles, risk of viral spread can be minimized.

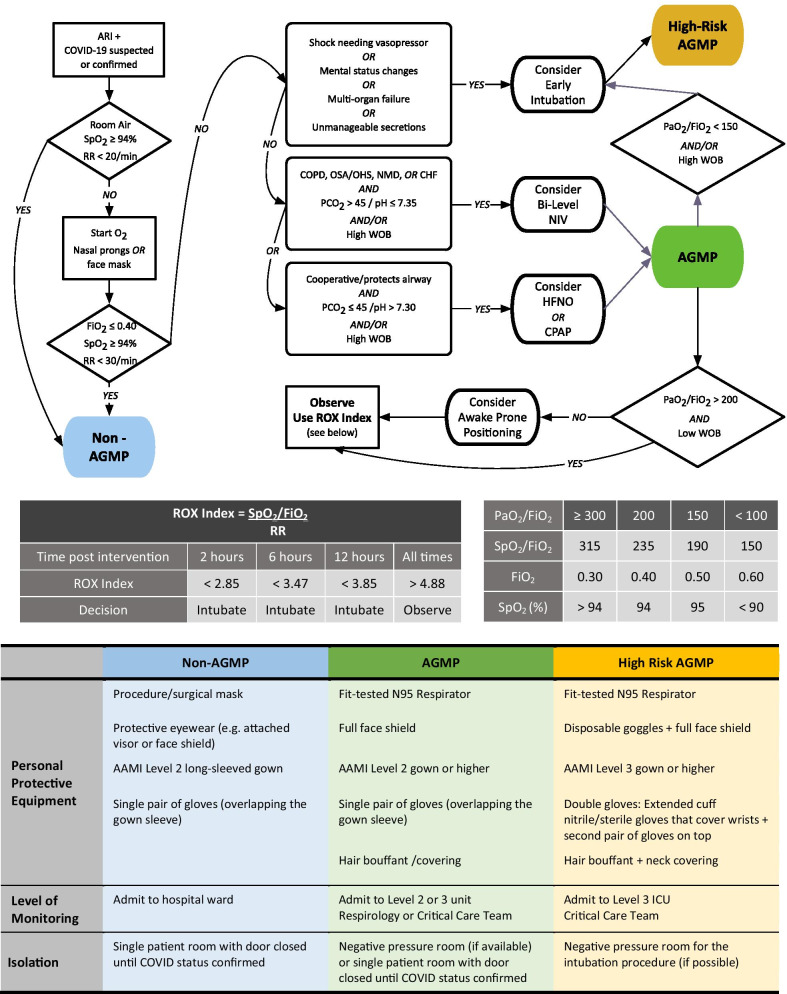

## Introduction

Preventing hospital transmission of COVID-19 is of utmost importance to avoid “accelerating the curve” during the pandemic. To that end, guidance issued early during the pandemic warned *against* use of aerosol-generating medical procedures (AGMP), such as non-invasive ventilation (NIV), continuous positive airway pressure (CPAP) and high flow nasal oxygen (HFNO), advocating instead for early intubation in patients with suspected/confirmed COVID-19 [1, 2]. In early 2020, as hospitals prepared for a surge in patients with COVID-19, this guidance was widely and rapidly adopted, resulting in confusion and some tragic results. In March, 2020, a patient presenting with an acute exacerbation of chronic obstructive pulmonary disease (COPD), who did not want intubation, died in the emergency room of our tertiary care academic centre, when he was denied NIV pending COVID-19 test result. Clearly, the edict against use of non-invasive respiratory support (NRS) was problematic. If all patients presenting to hospital with acute respiratory illnesses (ARI) were to undergo early endotracheal intubation (ETI), ICU capacity would quickly be exceeded. Furthermore, many patients presenting to hospital have common cardiorespiratory diseases for which NIV has proven efficacy, such as COPD and congestive heart failure (CHF) exacerbations, while others have advanced directives limiting life-extending technologies. To deny such patients, NRS options during the pandemic is neither rational nor ethical. Within months, experienced clinicians treating COVID-19 patients made pleas to reconsider the need for early, systematic intubation [3, 4]. Conversely, exposing healthcare providers (HCPs) to AGMP in patients potentially infected with the SARS-CoV-2 virus without due caution is reckless. How do we balance the need to care for COVID-19 suspect and positive patients and minimize risk of transmission while still providing evidence-based care to all hospitalized patients with ARI during this pandemic?

Available information on the risk and benefits of AGMP during the COVID-19 pandemic is rapidly evolving, with new observations and empirical data published daily, yet gaps remain between knowledge and practice. Knowledge translation tools are urgently needed to synthesize and transform the best available data into instructions that can be easily implemented by front-line HCPs at the bedside. At our tertiary care academic centre, spanning two hospitals serving a catchment area of 1 million people [5], we formed a multidisciplinary Ventilation Strategy for COVID-19 Working Group. Our objective, achieved with *rapid knowledge translation* of emerging literature, was to provide a comprehensive and timely narrative review of this topic and develop recommendations, educational materials and a decision-making algorithm to guide staff managing these patients. The key principles discussed here of mitigating risk of aerosolization, minimizing in-hospital viral transmission, managing acute respiratory failure non-invasively and evading patient self-inflicted lung injury will remain relevant for the next wave of COVID-19, the next influenza season or the next pandemic to come.

## Methods used to develop the knowledge translation tools

The multidisciplinary Ventilation Strategy for COVID-19 Working Group held its first virtual meeting 25 March 2020. Our aim was to minimize the risk of viral transmission with NRS strategies among various subgroups of patients and provide clear guidance to our front-line HCP on early management of patients with suspected or confirmed COVID-19. Our methodology included virtual discussion groups using Microsoft Teams^®^ and Zoom^®^ meeting software, evaluation of emerging published scientific literature, grey literature, Society (e.g., ESICM) webinars and newsletters, national/international health organization reports, as well as drawing upon email groups/personal communication with HCPs around the world to learn from their experience. Key articles were retrieved using OMNI Academic Search Tool (https://ocul.on.ca/omni/) which includes PubMed, Google Scholar, Scopus, MEDLINE and others, using search terms COVID-19; SARS-CoV-2; hypoxemic respiratory failure; and treatment. Time was critical. By 6 April 2020, we had our first documents approved by hospital leadership and available for use in our centre, which served our staff through the first wave of COVID-19. Updates were disseminated in April, July and September, 2020, as new information became available. Guidance issued around best practice in COVID-19 is based on low levels of evidence (case series, small observational studies, expert opinion, or extrapolated data) [6]. We share our approach with advisement that further research is required to answer several key questions, (see Recommendations for Clinical Practice and Future Research, Additional file 1) and encourage enrolment in randomized controlled trials where possible.

## Defining the risk of hospital transmission versus the risk of early intubation

The novel coronavirus SARS-CoV-2 has infected over 50 million people worldwide to date [7]; based on data from China, Europe and the USA, approximately 20% of those infected require hospitalization, and 3–7% require support for acute respiratory failure [8–12]. Recent data show that between 9 and 17% of COVID-19 cases are infected HCPs [13–15]. In northern Italy, 11.4% of HCPs working in respiratory units with patients undergoing AGMP tested positive for COVID-19 during a 2.5-month observation period [12]. The risk to HCP is not negligible; thus, their safety is paramount in the management of ARI throughout the pandemic.

Transmission of the SARS-CoV-2 virus is primarily through droplet spread [10]. These droplets (particles > 5–10 μm in diameter) are affected by gravity and may cause direct transmission from close contact or contribute to contamination of surfaces within 1.5–2.0 m, where the virus may remain active for hours to days [16, 17]. However, some events can generate aerosols composed of smaller virus-containing particles (< 5–10 μm) suspended in air. Until further data become available, it should be assumed that NRS measures are potentially AGMP. Dispersion distances for various treatment modalities have been described using human patient simulator technology to mimic different devices and severity of lung disease (Table 1) [18–22]. However, with careful attention to risk mitigation strategies, the maximum exhaled air distance may be reduced compared to conventional oxygen therapy (Table 1).

**Table 1:** Exhaled air dispersion distances during various treatments and conditions, with usual set-up

Condition	Distance (mm)	Mechanism of dispersion
Coughing [18]		
No mask	680	Forward jet
Wearing surgical mask	300	Sideway leakage
Wearing N95 mask	151	Sideway leakage
Simple oxygen mask [19]		
2 LPM	200	Lateral leakage from side vents
4 LPM	220	
8 LPM	300	
10 LPM	400	
HFNO [21] (normal lung condition*)		
10 L/min	65	
30 L/min	130	
60 L/min	172	
Displaced interface tube: 60 L/min	620	
NIV—CPAP [21]		
Swift FX nasal pillows: CPAP 5 cm H_2_O	207	
Swift FX nasal pillows: CPAP 20 cm H_2_O	332	
ResMed Quattro Air oronasal mask: CPAP 5 cm H_2_O	Negligible	
ResMed Quattro Air oronasal mask: CPAP 20 cm H_2_O	Negligible	Circular vent holes; no distinct jet
NIV—Bi-PAP/ total facemask [20]		
Respironics Full Face IPAP/EPAP: 10/5 cm H_2_O	618	Simulated for mild lung injury
Respironics Full Face IPAP/EPAP: 18/5 cm H_2_O	812	Simulated for mild lung injury
NIV—Bi-PAP/oronasal mask [22]		
Respironics Comfort 2 mask IPAP/EPAP: 10/4 cm H_2_O	650	
Respironics Comfort 2 mask IPAP/EPAP: 18/4 cm H_2_O	850	
Respironics Image 3 mask + Whisper Swivel		
IPAP/EPAP: 10/4 cm H_2_O	950	
IPAP/EPAP: 18/4 cm H_2_O	> 950	Diffuse dispersion
NIV—Bi-PAP/ helmet [20]		
Sea-Long helmet IPAP/EPAP: 12/10 cm H_2_O	150	Dispersion through neck interface
Sea-Long helmet IPAP/EPAP: 20/12 cm H_2_O	230	
StarMed CaStar R helmet	Negligible	Better neck seal using air cushion

All studies by David Hui

Airflow was marked with intrapulmonary smoke for visualization using a human patient stimulator (HPS) to mimic different devices and severity of lung injury

*Less exhaled distances with more severe lung injury at all flows during HFNO

Avoidance of NRS in patients with suspected/confirmed COVID-19 in favour of early endotracheal intubation (ETI) as first-line therapy carries risk of morbidity to patients, including immobilization, disuse diaphragmatic atrophy, ventilator-associated infections, and potential for long-term physical and neurocognitive dysfunction [23], with risk of overwhelming ICU and ventilator capacity. Thus, a strategy is required to identify and safely manage patients likely to benefit from NRS while protecting HCP from risk of contagion through AGMP, and to identify those patients likely to require early ETI, protecting them from risk of increased mortality associated with delay of inevitable intubation [24].

## Clinical management of ARI during the pandemic

COVID-19 should be suspected in patients presenting with an acute or acute on chronic respiratory illness. In addition to causing de novo ARI, the virus may also cause worsening of underlying cardiorespiratory disease with an acute exacerbation of COPD or CHF, or respiratory failure in the setting of pulmonary hypertension, obstructive sleep apnea (OSA)/obesity hypoventilation syndrome (OHS), or neuromuscular disease. Patients with acute on chronic respiratory failure may or may not have concomitant COVID-19 infection, but appropriate precautions should be taken until confirmed negative by testing. After donning appropriate personal protective equipment (PPE), isolating the patient from other patients, and sending a nasopharyngeal swab viral polymerase chain reaction (PCR) for SARS-CoV-2, the next step is to determine the most appropriate respiratory support.

**Fig.1:**
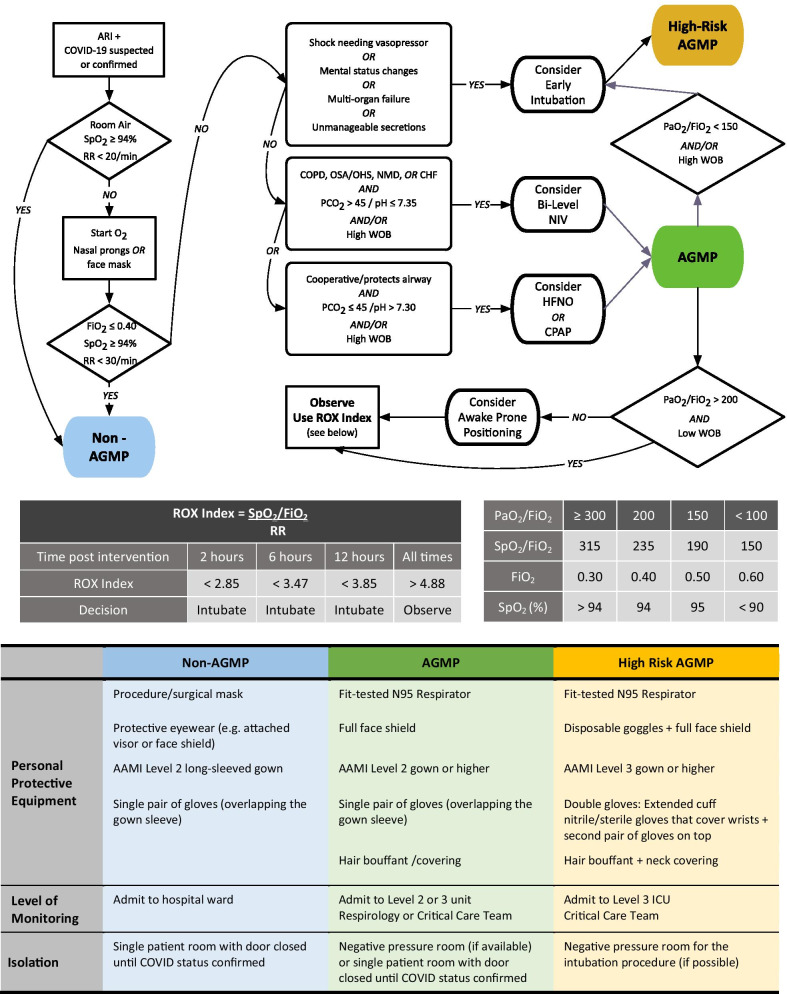
**a** Acute respiratory illness (ARI) early management decision algorithm (COVID-19). The ARI decision algorithm guides determination of the level of support required for the hypoxemic patient, and patient factors that determine appropriateness for NIV, HFNO, awake prone positioning and intubation. **b** Personal protective equipment, isolation and level of monitoring required for various treatments and conditions during COVID-19. This table accompanies **a** and outlines the PPE, isolation strategy and level of monitoring required for non-AGMP, AGMP and high-risk AGMP care. *ARI *acute respiratory illness, *RR, *respiratory rate, *AGMP* aerosol generating medical procedure, *COPD *chronic obstructive pulmonary disease, *OSA *obstructive sleep apnea, *OHS *obesity hypoventilation syndrome, *NMD *neuromuscular disease, *CHF* congestive heart failure, *WOB* work of breathing, *HFNO* high flow nasal oxygen, *NIV* non-invasive ventilation, *Bi-PAP* bi-level positive airway pressure, *CPAP* continuous positive airway pressure. *AAMI* Association for the Advancement of Medical Instrumentation, *PPE* personal protective equipment, *ICU* intensive care unit

Figure 1 represents a summary of recommendations as a decision algorithm (1A) and accompanying table (1B) for the early management of ARI during the COVID-19 pandemic. The algorithm is based on upholding best-evidence guidelines for non-COVID patients, and emerging evidence and worldwide clinical experience with COVID-19 during the pandemic. The purpose of this tool is to identify and categorize patients into three groups based on their likelihood of requiring non-AGMP support, AGMP or high-risk AGMP (intubation) as first-line therapy, so that patients can be admitted to the appropriate area within the hospital with the necessary level of expertise and appropriate precautions taken by HCPs. The decision algorithm was designed to be a pragmatic, easily applied bedside tool, and hence, we used pulse oxygen saturation (SpO_2_) and fraction of inspired oxygen (FiO_2_) values, but provided relative PaO_2_/FiO_2_ values for reference.

Patients with elevated respiratory rate and SpO_2_ < 94% on room air need oxygen applied by nasal prongs or face mask [6]. Patients without distress who are able to maintain a SpO_2_ ≥ 94% on a FiO_2_ ≤ 0.40 may be admitted to a hospital ward single-patient room and observed [6]. Patients with persistent elevated respiratory rate and moderate to severe hypoxemia require further assessment to determine whether early ETI will be necessary, or if NRS is appropriate.

In an effort to balance the risks of invasive mechanical ventilation with deleterious consequences of delayed intubation, we recommend consideration of intubation as the initial approach for patients with mental status changes (e.g., agitation or obtundation), shock requiring vasopressors, multi-organ failure (e.g., acute kidney injury requiring renal replacement therapy) or unmanageable secretions accompanying hypoxemia or acidosis. Such patients are not appropriate for NRS [25, 26], and early intubation facilitates safe airway management and protective lung ventilation which would not be possible with the spontaneous-breathing patient [23].

Co-operative patients with single system respiratory failure who do not require prompt intubation may be managed with NRS, but must be monitored closely for response to treatment. Both NIV (bi-level positive airway pressure) and CPAP should remain the treatment of choice as per usual indications: CPAP for CHF and OSA, and NIV for COPD exacerbations, neuromuscular disease or OHS complicated by hypercapnic respiratory failure [25]. If NIV is being considered for acute on chronic hypercapnic respiratory failure, this should be initiated at hospital admission. Do not prevent NIV use where previously appropriate prior to the COVID-19 pandemic.

Patients with de novo hypoxemic respiratory failure may be considered for HFNO [26] or CPAP (preferably by helmet), if HFNO is not available. Potential candidates for HFNO or CPAP should be alert, cooperative, able to protect their airway, with acceptable ventilation (pH > 7.30). Work of breathing should decrease with NRS measures and may be assessed by palpation of the sternomastoid muscle, detection of phasic contraction [27] and/or a reduction in an elevated serum lactate produced by fatiguing respiratory muscles [28]. HFNO and CPAP can support both oxygenation and ventilation by reducing work of breathing for patients with hypoxemia and dyspnea with presumed COVID-19 pneumonia [29, 30]. In Lombardy Italy, where numbers of COVID-19 patients surpassed ICU capacity, necessitating NRS in specially developed Respiratory COVID Units, ETI was avoided in approximately 2/3 of patients without increasing the relative risk of death [12]. However, available best practice guidelines [29–31] suggest NRS should not be used for severe hypoxemic respiratory failure with high respiratory rate/high work of breathing not relieved with support [32], or a trajectory that suggests that invasive ventilation is inevitable. Patients with high respiratory rate or effort in the setting of acute lung inflammation are at risk of exacerbating the acute lung injury by means of hyperventilation or high transpulmonary pressures, termed “patient self-inflicted lung injury” (P-SILI) [33, 34]. Furthermore, if NRS does not reduce respiratory effort, patients may fatigue [32] and/or deteriorate precipitously. In such circumstances, patients should be intubated and transitioned to invasive ventilation without delay. Although intubation is the preferred option for patients failing to meet targets on HFNO, it is acceptable to use NIV for patients with restricted resuscitation goals that preclude intubation.

After HFNO or CPAP initiation, patients *may* be encouraged to assume the prone position, particularly if the PaO_2_/FiO_2_ ratio is below 200. The suggestion for a trial of awake prone positioning during NRS is based on physiologic benefit [35] and extrapolation from non-COVID studies rather than proven clinical outcomes in COVID-19 patients. Ventilation in prone position reduces mortality in patients with ARDS receiving invasive mechanical ventilation [36, 37] and improves oxygenation in awake, spontaneously breathing patients with moderate to severe ARDS receiving oxygen therapy by HFNO or NIV [38, 39]. Although small case series of spontaneously breathing and NIV-assisted COVID-19 patients have recently described feasibility, tolerance and safety with improvement in oxygenation, larger randomized controlled trials are needed to determine if it improves outcomes [40–43]. In our experience, patients are able to pronate themselves but may need assistance adjusting their HFNO or NIV interface with turns. Although less complicated and labour-intensive than prone positioning in unconscious patients, potential risks and barriers include patient discomfort, nausea, increased leak from the interface, and nurse and respiratory therapist time to assist. If considered, prone positioning should be implemented early after hospital admission in patients fitting selection criteria (i.e., cooperative, able to protect airway, with low work of breathing) [44]. Thoracic CT and ultrasound findings [45] in COVID-19 are varied, but prone positioning may best help those with dorsal lung region ground glass consolidation and/or atelectasis through more homogenous lung inflation and improved ventilation-perfusion matching (i.e., when dorsal regions become nondependent) [23]. Encourage patients to accrue a total of 8 to 16 h per 24 h in the prone position, especially over the first 24–48 h. Ensure patients have access to oral suction, the means to contact the nurse (e.g., call bell, baby monitor), have continuous SpO_2_ monitoring and frequent assessment of respiratory rate and work of breathing. Aborting prone positioning in favour of intubation should not be delayed if failing HFNO/NIV.

The respiratory rate-oxygenation (ROX) index, developed to identify patients at high risk for needing intubation while on HFNO [46], may help guide intubation decision-making [45]. The ROX index is calculated as:

$${\text{ROX}}\;{\text{index }} = \frac{{ {\text{SpO}}_{{2}} /{\text{FiO}}_{{2}} }}{{{\text{Respiratory}}\;{\text{Rate}}}}$$

Previously healthy patients with normal lung compliance and cardiac output are likely to tolerate a lower SpO_2_ without significant distress. A ROX index ≥ 4.88 is reassuring, and such patients can continue to be observed. Figure 1a shows the ROX index thresholds at various time points which should prompt a change in management and consideration of intubation. The trend in ROX index over time may be as indicative as the absolute value, as the ROX index should improve over time. While validated for use during HFNO [46], the ROX index has not been studied for its predictive value in COVID-19 specifically and should not supplant clinical exam or clinical judgement. Furthermore, patients who develop acidosis, confusion, changes in mentation or are unable to manage their secretions should be intubated and invasively ventilated using a lung protective strategy.

Finally, patients undergoing NRS should be cared for in a monitored setting with well-trained staff accustomed to use and titration of these modalities (Fig. 1b). At our institution, our Respirology Service (led by staff pulmonologist with resident house staff) or Critical Care Outreach Team (a Rapid Response Team led by staff intensivist with specially trained ICU registered nurse and registered respiratory therapist) must be consulted to manage all patients on NRS, with patients admitted to a Respiratory Unit, ward or ICU where bedside staff are appropriately trained. Both pre-COVID and COVID-19 experience support the association between admission to the appropriate setting with team expertise and better outcomes for NRS [3, 12].

## Preventing hospital transmission of COVID-19 through isolation and PPE

To reduce hospital transmission, environmental control and appropriate PPE must be considered when managing patients. Suspected or confirmed COVID-19 patients requiring hospital admission and undergoing AGMPs should be admitted to a negative pressure room, if available, otherwise, single-patient rooms (with door closed). Negative pressure rooms within the Emergency Department or ICU may be reserved for patients requiring ETI on arrival, as the intubation procedure is a high-risk AGMP. Rapid sequence intubation should be performed by the most experienced person with a limited number of HCPs in the room [1, 2, 47]. Where available, specialized “intubation teams” of highly experienced HCPs may perform all intubations in COVID-19 suspect/ confirmed cases [47, 48]. A hydrophobic filter should be interposed between the facemask and breathing circuit. After the intubation procedure is complete, patients receiving invasive mechanical ventilation through a closed circuit may be moved out of negative pressure rooms and cohorted according to COVID-19 status. The number of air exchanges per hour in the room will determine the length of time to clear the air of aerosolized particles after completion of an AGMP within the room.

If a patient develops symptoms suggestive of COVID-19 while in hospital, the patient should be transferred to a single patient or negative pressure room for AGMP with appropriate PPE used. CT scan may improve diagnostic sensitivity, particularly in the early phase of infection where nasopharyngeal swab PCR may be falsely negative [49]. If test results confirm the patient is COVID-19 negative, no further action is needed. The area(s) will need thorough cleaning as the virus does survive on stainless steel and plastic for up to 48 h [16].

Detailed guidelines for PPE required during AGMPs and recommendations for optimizing the supply of PPE during the COVID-19 pandemic are available [17, 50–52]. As described by Lockhart et al., [17] we recommend a three-tiered approach to PPE, as shown in Fig. 1b. Care with donning and doffing of PPE is crucial and should be reviewed in instructional videos [53] and practiced under supervision.

## Technical aspects: mitigation techniques to reduce exhaled droplet dispersion

**Fig. 2:**
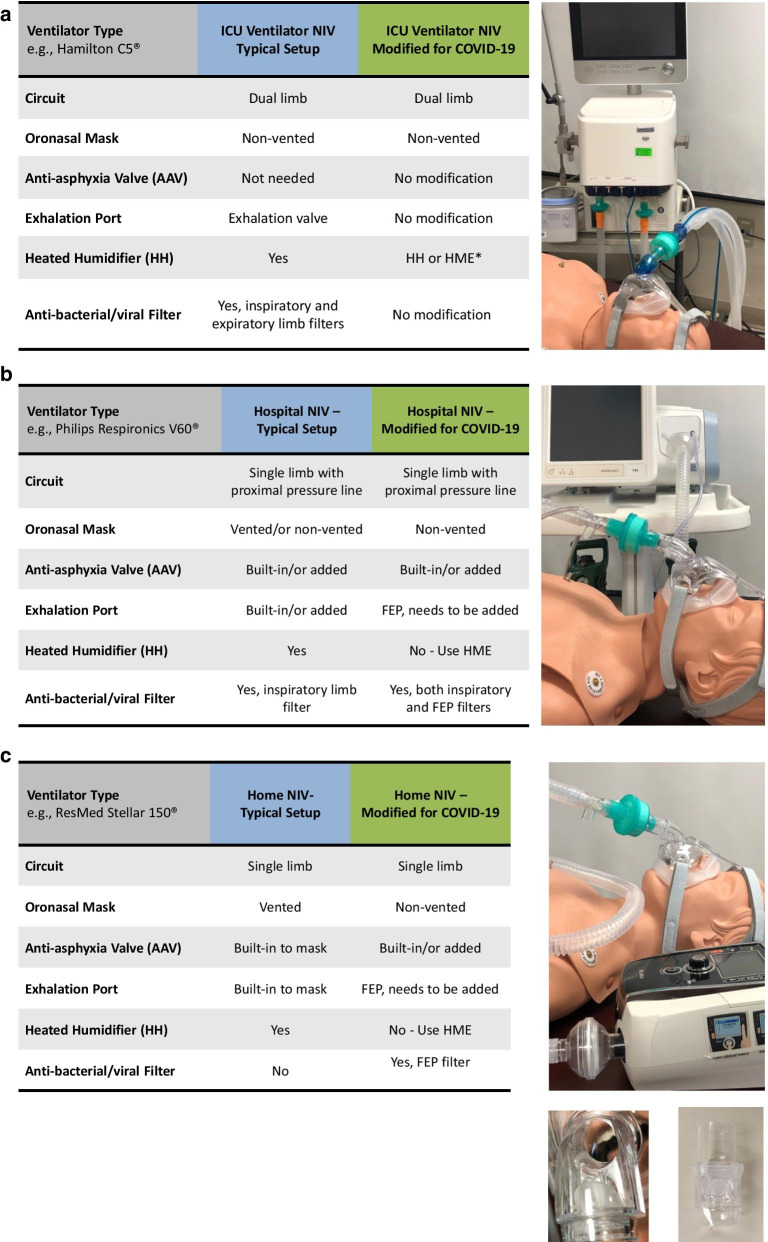
**a** Modified ICU NIV. Pictured is the Hamilton C5^®^ ventilator with dual limb circuit but without heated humidifier. Non-vented mask; combined anti-bacterial/viral filter/HME; and flow sensor lines. Filters at inspiratory and expiratory ports. **b** Modified Hospital NIV. Pictured is the Philips Respironics V60^®^ ventilator with single limb circuit but without heated humidifier. Non-vented mask with anti-asphyxia valve; combined anti-bacterial/viral filter and HME; distal exhalation port; and proximal pressure line. Second filter at inspiratory port. **c** Modified Home NIV. Pictured is the ResMed Stellar 150^®^ bi-level ventilator with single limb circuit but without heated humidifier. Non-vented mask with anti-asphyxia valve; combined anti-bacterial/viral filter/HME; and distal exhalation port. Second filter at inspiratory port. Oxygen port at rear of device. Insert details the anti-asphyxia valve. *NIV* non-invasive ventilation, *HH* heated humidifier, *FEP* filtered exhalation port, *HME* heat and moisture exchanger, *AAV* anti-asphyxia valve; *determined by local practice

**Fig. 3:**
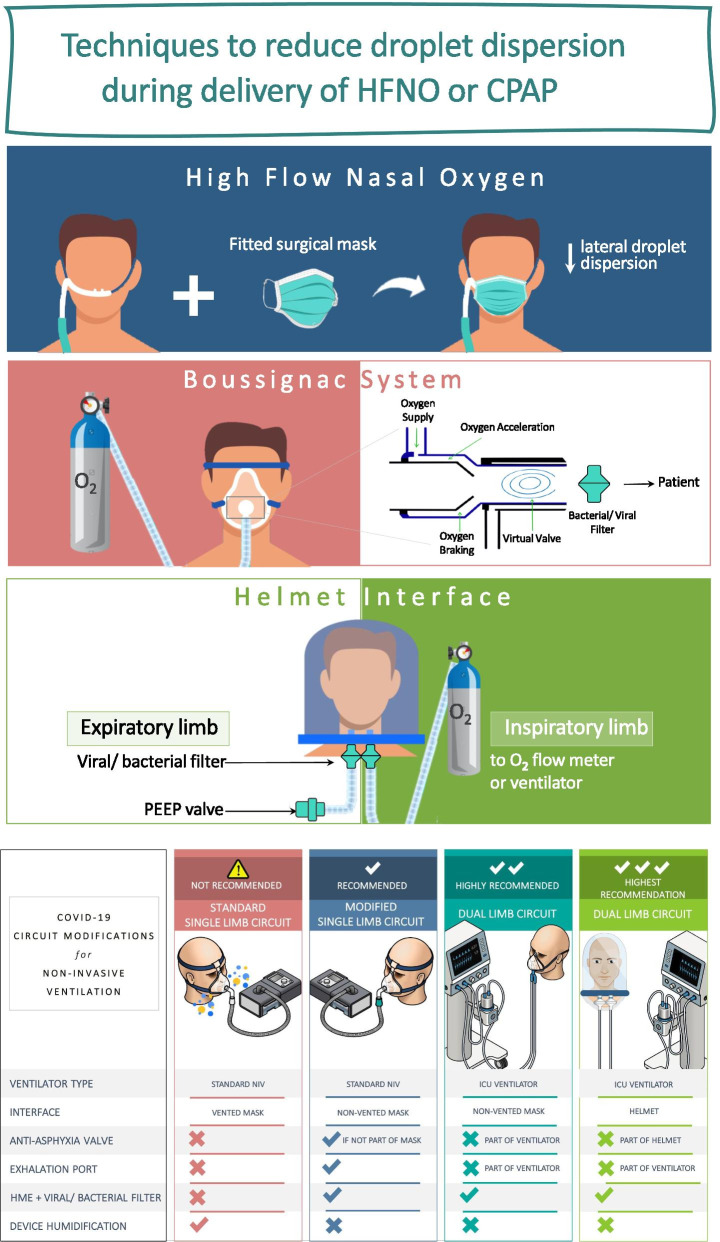
**a** Infographic. Techniques to reduce droplet dispersion during HFNO and CPAP. Pictorial representation of techniques to reduce droplet dispersion during aerosol-generating medical procedures. **b** Infographic. COVID-19 Circuit Modifications for Non-Invasive Ventilation. Pictorial representation of circuit modifications for NIV use during the COVID-19 pandemic

Respiratory care exposes HCPs to respiratory droplets. Mitigation techniques can substantially reduce droplet deposition during NRS. Figure 2 is a picture guide demonstrating device modifications for NIV [54–56], and Fig. 3 is an infographic summarizing risk mitigation techniques for use during AGMPs.

### High flow nasal oxygen

HFNO is an open-interface high flow oxygen delivery system which may be better tolerated than oxygen by nasal prongs or mask to treat hypoxemia due to COVID-19 pneumonia. Mitigation of droplet transmission associated with HFNO may be achieved using a properly fitting surgical facemask over the HFNO cannula to reduce lateral droplet dispersion [57] (Fig. 3a). When using HFNO, deliver 40 to 60 L/min of gas flow and lowest FiO_2_ possible to maintain SpO_2_ in the range of 92–96% [6].

### Boussignac CPAP system

The Boussignac CPAP system is a simple method that works using the venturi principle with wall oxygen flow. A ventilator/CPAP device is not required [58, 59]. With the Boussignac system, air or oxygen is injected through the micro-channels in the wall of the plastic tube. As gas molecules accelerate through the channels and enter the cylinder, a virtual valve is created, resulting in continuous positive airway pressure (Fig. 3A). Oxygen flow of 8 L/min creates a CPAP pressure of 3 cmH_2_O; 15 L/min results in 5 cmH_2_O; and 23 L/min (or flush) provides 10 cmH_2_O of pressure. A bacterial/viral filter should be inserted between the mask and the Boussignac valve.

### Helmet CPAP system

CPAP may be delivered via the helmet interface with the inspiratory limb connected to a free flow oxygen system and the expiratory limb connected to a positive end-expiratory pressure (PEEP) valve (Fig. 3a) [60]. Set oxygen flow at 50–60 L/minute to ensure carbon dioxide (CO_2_) washout from the helmet; FiO_2_ may be adjusted but do not set flow lower than 50 L/minute to avoid CO_2_ rebreathing [60]. Alternatively, the helmet may be connected to a ventilator to deliver CPAP or bi-level pressures.

### Non-invasive ventilation

Where experience exists, delivery of NIV using a helmet interface may offer reduced droplet spread [61], improved patient tolerance [61] and efficacy [62] over an oronasal mask. The helmet is connected to an ICU ventilator using conventional respiratory circuitry joining two port sites to allow inspiratory and expiratory flow. High flow and short inspiratory time are necessary to pressurize the helmet rapidly. As shown in Table 1, second generation helmets have negligible exhaled air dispersion due to a better seal at the neck [20].

Where helmets and/or expertise utilizing them are not available, an oronasal non-vented mask (rather than nasal interface) should be used. Proper mask fitting and seal is important for oronasal non-vented masks, to minimise droplet dispersion and maximize effectiveness. Where possible, use a ventilator with a dual limb circuit plus heat and moisture exchanger (HME) filter with a non-vented mask (no anti-asphyxia valve is needed) (see Fig. 2a). Sequence of actions: put NIV interface on patient; then turn ventilator on; and turn ventilator off before removing NIV interface. If possible, do not use the device humidifier. Patients will require enhanced mouth care for dryness given increased airflow without humidification. If the patient has secretions with strong cough or is expected to require NIV for a prolonged period, device humidification may be needed and may be used with a dual limb circuit. Increased risk of aerosolization of virus-containing water droplets must be weighed against the risk of mucous plugging [63].

A single-circuit bi-level ventilator may need to be used if a dual circuit ventilator is not available or not tolerated. In this case, use a fitted oronasal non-vented mask plus anti-asphyxia valve with combined HME-viral/bacterial filter plus exhalation port. An anti-asphyxia valve is mandatory for use with a non-vented mask. The anti-bacterial/viral filter should be placed in the circuit between the mask and the exhalation port (see Fig. 2b). Anti-bacterial/viral filters should be changed every 24 h or sooner if soiled as this may increase resistance to flow. Blocked filters can be mistaken for clinical deterioration, and this issue is remedied by changing filters. An external humidifier should not be used.

Initial prescription for single-circuit bi-level ventilation for de novo ARI: quick rise time (~ 200 ms); high trigger; low cycle; expiratory positive airway pressure (EPAP) 8–12 cm H_2_0; and minimal pressure support (inspiratory positive airway pressure, IPAP ≤ 5 cm H_2_0 above EPAP). Target and monitor for Vt ~ 4–7 mL/kg ideal body weight and a SpO_2_ ≥ 92–96% [64] using the lowest FiO_2_ possible.

### Home mechanical ventilation patients

Patients receiving mechanical ventilation at home (e.g., neuromuscular disease) may present to the emergency room with/without respiratory symptoms using a single-circuit bi-level ventilator and vented mask and/or cough assist device in the community. Continuation of this support is essential to their survival. Home NIV circuit modifications are required using an oronasal non-vented mask with anti-asphyxia valve and expiratory port with anti-bacterial/viral filter (see Fig. 2c). A variety of circuit modifications can be used [56]. Otherwise, use their home ventilator and prescription, care for them in a single room, and staff should wear AGMP PPE (including N95 mask) while in the patient room.

## Limitations

Emerging data on use of NRS in COVID-19 are limited to observational studies demonstrating feasibility and physiologic benefits rather than trials evaluating clinically important outcomes. Furthermore, due to the urgency of publishing, heterogeneity in study design and reporting of data make comparisons across centres problematic. Notwithstanding, the knowledge translation tools we developed are based on best available evidence and were utilized in our hospitals with excellent uptake and acceptance by staff.

## Conclusion

During the COVID-19 pandemic, patients may present with various etiologies of ARI, requiring differing support levels for oxygenation and ventilation. The evidence for NRS versus early ETI in COVID-19 is still evolving. Despite limitations of existing data, HCPs must still act with the best knowledge available. In that context, it is prudent to suspect COVID-19 infection in all patients with respiratory symptoms and/or hypoxemia until ruled out, but suspicion of COVID-19 does not necessitate early intubation in all patients. Selected patients may be managed with NRS provided appropriate precautions are taken to mitigate nosocomial transmission, patients are closely monitored, and hypoxemic patients proceed to prompt intubation when necessary.

## Supplementary information

**Additional file 1.** Recommendations for Clinical Practice and Future Research.

## Data Availability

Not applicable.

## Abbreviations

COVID-19: Coronavirus disease 2019
AGMP: Aerosol-generating medical procedures
ROX index: Respiratory rate-oxygenation index
NIV: Non-invasive ventilation
CPAP: Continuous positive airway pressure
HFNO: High flow nasal oxygen
COPD: Chronic obstructive pulmonary disease
NRS: Non-invasive respiratory support
ARI: Acute respiratory illness
ICU: Intensive care unit
CHF: Congestive heart failure
HCP: Healthcare provider
SARS-CoV-2: Severe acute respiratory syndrome-related coronavirus 2
P-SILI: Patient self-inflicted lung injury
ETI: Endotracheal intubation
PPE: Personal protective equipment
PCR: Polymerase chain reaction
ARDS: Acute respiratory distress syndrome
CT: Computed tomography
OSA: Obstructive sleep apnea
OHS: Obesity hypoventilation syndrome
SpO_2_: Saturation of haemoglobin with oxygen as measured by pulse oximetry
FiO_2_: Fraction of inspired oxygen
PaO_2_: Partial pressure of oxygen (arterial)
PEEP: Positive end expiratory pressure
CO_2_: Carbon dioxide
HME: Heat and moisture exchanger
EPAP: Expiratory positive airway pressure
IPAP: Inspiratory positive airway pressure
Vt: Tidal volume
