# Supplementary material for: Knowledge translation tools to guide care of non-intubated patients with acute respiratory illness during the COVID-19 Pandemic
Source: Crit Care. 2021 Jan 8;25:22. doi: 10.1186/s13054-020-03415-2 (PMC7791165; doi:10.1186/s13054-020-03415-2)
Supplement: Supplementary file 1 — Additional file 1. Recommendations for Clinical Practice and Future Research. [file 13054_2020_3415_MOESM1_ESM.docx]

**Additional File 1. List of Recommendations for Clinical Practice and Future Research**

| **Recommendations for Clinical Practice** | **Recommendations for Future Research** |
| --- | --- |
| Patients admitted to hospital with ARI should be tested for SARS-CoV-2, isolated, placed under appropriate precautions and observed. |  |
| PPE must be appropriate to conditions of treatment (non-AGMP, AGMP, high-risk AGMP) |  |
| Non-invasive support measures such as NIV and HFNO should not be denied for conditions where previously proven effective. |  |
| Prone position may be used to improve oxygenation in awake, spontaneously breathing patients on HFNO or NIV. | Does prone positioning of non-intubated patients reduce rates of intubation and/or mortality? |
| Patients with COVID-19 are at risk of P-SILI and must be monitored closely; the ROX index may help to guide intubation decision | What are the predictors of P-SILI in COVID-19? Does ROX index below threshold values indicate “safe” transpulmonary pressures? |
| Non-invasive measures such as HFNO, CPAP may be used to support oxygenation and reduce work of breathing in patients with COVID-19, provided mitigation techniques are employed to reduce droplet dispersion. | Do non-invasive measures (HFNO/ NIV) reduce intubation rates and/or mortality?  Do non-invasive measures delay intubation and increase mortality?  What results in lower mortality: early intubation and lung protective ventilation vs. non-invasive measures to support and enable awake, spontaneous breathing? |
| For HFNO, place a surgical mask over the nasal cannula.  For CPAP, use of helmet interface is preferred. If not available, use a properly fitted oronasal mask or Boussignac CPAP device, with a bacterial/viral filter inline.  For NIV, a dual limb circuit with helmet interface is preferred. If helmet is not available, use an oronasal non-vented mask and AAV. Use HME rather than device humidification if possible. | What are the exhaled air/droplet dispersion distances with these mitigation techniques? Can further modifications be made to interface or circuit to further mitigate droplet dispersion? |
